# RAG-1 and Ly6D Independently Reflect Progression in the B Lymphoid Lineage

**DOI:** 10.1371/journal.pone.0072397

**Published:** 2013-08-30

**Authors:** Qingzhao Zhang, Brandt L. Esplin, Ryuji Iida, Karla P. Garrett, Zhixin L. Huang, Kay L. Medina, Paul W. Kincade

**Affiliations:** 1 Immunobiology & Cancer Program, Oklahoma Medical Research Foundation, Oklahoma City, Oklahoma, United States of America; 2 Department of Microbiology and Immunology, University of Oklahoma Health Sciences Center, Oklahoma City, Oklahoma, United States of America; 3 Department of Immunology, Mayo Clinic, Rochester, Minnesota, United States of America; New York University, United States of America

## Abstract

Common lymphoid progenitors (CLPs) are thought to represent major intermediates in the transition of hematopoietic stem cells (HSCs) to B lineage lymphocytes. However, it has been obvious for some time that CLPs are heterogeneous, and there has been controversy concerning their differentiation potential. We have now resolved four Flt3^+^ CLP subsets that are relatively homogenous and capable of forming B cells. Differentiation potential and gene expression patterns suggest Flt3^+^ CLPs lacking both Ly6D and RAG-1 are the least differentiated. In addition to B cells, they generate natural killer (NK) and dendritic cells (DCs). At the other extreme is a subset of the recently described Flt3^+^ Ly6D^+^ CLPs that have a history of RAG-1 expression and are B lineage restricted. These relatively abundant and potent CLPs were depleted within 48 hours of acute *in vivo* estrogen elevation, suggesting they descend from hormone regulated progenitors. This contrasts with the hormone insensitivity of other CLP subsets that include NK lineage progenitors. This progenitor heterogeneity and differentiation complexity may add flexibility in response to environmental changes. Expression of RAG-1 and display of Ly6D are both milestone events, but they are neither synchronized nor dependent on each other.

## Introduction

Relationships between hematopoietic stem cells (HSCs) and B lineage lymphocytes have long been studied as a model for differentiation and as one that is informative about several diseases. For instance, the position of transformed cells in a maturation sequence has prognostic value, and pinpointing developmental arrests is helpful in studying immunodeficiencies. In general, the process yields cells with progressively fewer differentiation options, and it would seem possible to describe it in a simple diagram. However, accumulating information has revealed lymphocyte formation to be more dynamic and complex than previously imagined [1]. For example, HSCs are surprisingly heterogeneous, and some subsets are more prone to generate lymphocytes after transplantation than others [2], [3].

The lymphopoietic progenitors that arise from HSC are also heterogeneous [4]-[13], and more information would help arrange subsets in a hierarchy. The goal of this study was to use three previously known parameters together to chart major differentiation pathways. Specifically, display of Flt3, a history of RAG-1 locus activation and expression of Ly6D are all well-established differentiation milestones in the B lymphocyte lineage [7], [12]-[15]. Models based on those parameters have allowed subdivision of lymphoid progenitors, and helped to position B lineage progenitors in a sequence [5], [8], [9]. Yet, it has been unclear if there is a single, strict order to these events.

Up-regulation of Flt3 on lineage marker negative (Lin^-^), c-Kit^Hi^, Sca-1 bearing bone marrow cells signals loss of erythroid and megakaryocytic differentiation potential, and Lin^-^ c-Kit^Hi^ Sca-1^+^ Flt3^Hi^ cells are often referred to as lymphoid primed multipotent progenitors (LMPPs) [5], [12]. The same fraction includes cells that are very potent with respect to lymphocyte production and have diminished potential to make non-lymphoid cells [5], [12], [16]. Our lab refers to them as early lymphoid progenitors (ELPs) [5], [17]. Viable ELPs sorted on the basis of RAG-1 reporters require 10 days to generate CD19^+^ B lineage lymphocytes in culture, while otherwise similar c-Kit^Lo^ cells do so more rapidly [5], [18]. Thus, time required for differentiation is a measure of the positions of progenitors relative to each other.

Kondo and colleagues discovered a major intermediate in lymphopoiesis, and they termed this Lin^-^ IL-7Rα^+^ Sca-1^/lo+^ c-Kit^Lo^ fraction common lymphoid progenitors (CLPs) [6]. While this represented a major advance, the same name has been used to describe progenitors with different properties. For example, Lin^-^ IL-7Rα^+^ CD93^+^ Sca-1^Lo^ [19]; Lin^-^ Il-7Rα^+^ c-Kit^+^ CD24^Lo^ CD93^Hi^ CD43^Lo^ [20]; Lin^-^ IL-7Rα^+^ c-Kit^Lo^ Sca-1^Lo^ Thy1.1^+^ Flt3^+^ [8] and Lin^-^ CD27^+^ Flt3^+^ IL-7Rα^+^ c-Kit^Hi/lo^ Ly6D^-^ [9] have all been designated CLPs. Notably, c-Kit and Sca-1 were not used in as gating criteria in two recent characterizations of CLPs [9], [11]. Absence of lineage associated markers and display of IL-7Rα were the only features common to all of these descriptions of CLPs. Adding to the confusion, detection of low level expression of IL-7Rα improved with more sensitive flow cytometry, such that small numbers of c-Kit^Hi^ LMPPs/ELPs would now be considered CLPs by some definitions [21]. CLPs can be subdivided by current or past RAG-1 expression as well as by their utilization of the Ig gene recombination machinery [5], [13]-[15]. In addition to defining LMPPs/ELPs, expression of Flt3 helps to define CLPs with more restricted B, T and NK lymphoid potential [8]. Finally, Ly6D is useful inasmuch as its expression correlates with absolute restriction to the B lymphocyte lineage [9]. Therefore, it should be helpful to know the relative value of these sort parameters when used alone or in combinations.

The existence of lymphopoietic cells with greatly reduced, but not absent potential to generate non-lymphoid cells has been substantiated with fate mapping systems [14], [22]. Thus, common lymphoid progenitors in bone marrow may normally be used to replenish B and T lymphocytes, even though alternate differentiation routes can be used in some circumstances.

We will conclude that the actual sequence of marker expression by CLPs in bone marrow is not rigid. Our new findings confirm previous reports that expression of Ly6D and RAG-1 are each highly correlated with restricted B lineage potential [9], [23]. However, the two markers were particularly effective when used in combination and a Ly6D^+^ RAG-1^+^ subset was identified that is extremely potent in generating B lineage lymphocytes. The abundance and maturity of these particular progenitors relative to other Flt3^+^ CLP subsets suggests they must represent major intermediates in B cell production. Furthermore, their numbers can be regulated by sex steroids.

## Results

### Resolution of CLP subsets

**Figure 1:**
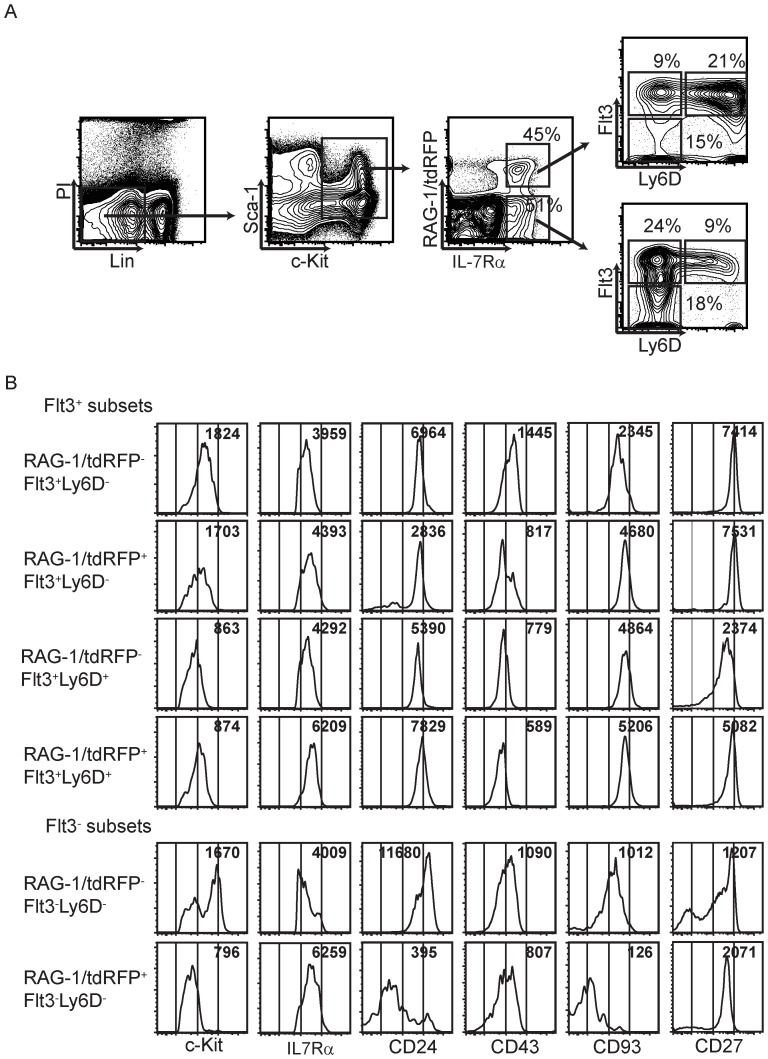
Resolution and characterization of CLP subsets in RAG1/Red fate mapping mice. (**A**) The dot plot profile displays the gating strategy of CLPs and the six subsets. The numbers indicate percentages of total CLPs that were gated as Lin^-^ c-Kit^+^ IL7R-α^+^. (**B**) The histogram profiles demonstrate densities of surface markers expressed on each CLP subset, and the numbers are average mean fluorescence intensities (MFI) calculated from three experiments. All data shown are representative of at least three independent experiments.

There are at least five definitions for CLPs [6], [8], [9], [19], [20]. Most, if not all of these populations are functionally heterogeneous [4], [7], [9]-[11], [13], so we used an inclusive strategy for their identification and isolation. Sca-1 was omitted as a gating parameter to be consistent with two recent studies [6], [9], [11] while expression of c-Kit was used to exclude contamination with a substantial Lin^-^ c-Kit^-^ Sca-1^+^ Flt3^+^ population [24], [25]. We then used three informative parameters to sub-divide Lin^-^ c-Kit^+^ Sca-1^+^ IL-7Rα^+^ CLPs into six subsets (Figure 1A). Flt3 is up-regulated as HSCs give rise to very primitive LMPP/ELPs [5], [12], [16]. Flt3 can also be used to sub-divide CLPs, marking cells capable of generating B and T lineage lymphocytes [8]. As previously shown, CLPs from RAG-1/Red mice are divisible into two approximately equal subsets differing with respect to a history of RAG-1 expression [14]. We found that both RAG-1/tdRFP positive and negative categories of CLPs include Flt3^+^ cells. As a final separation parameter, Ly6D discriminates B lineage restricted CLPs, and the majority of Ly6D^+^ CLPs express Flt3 [9].

**Figure 2:**
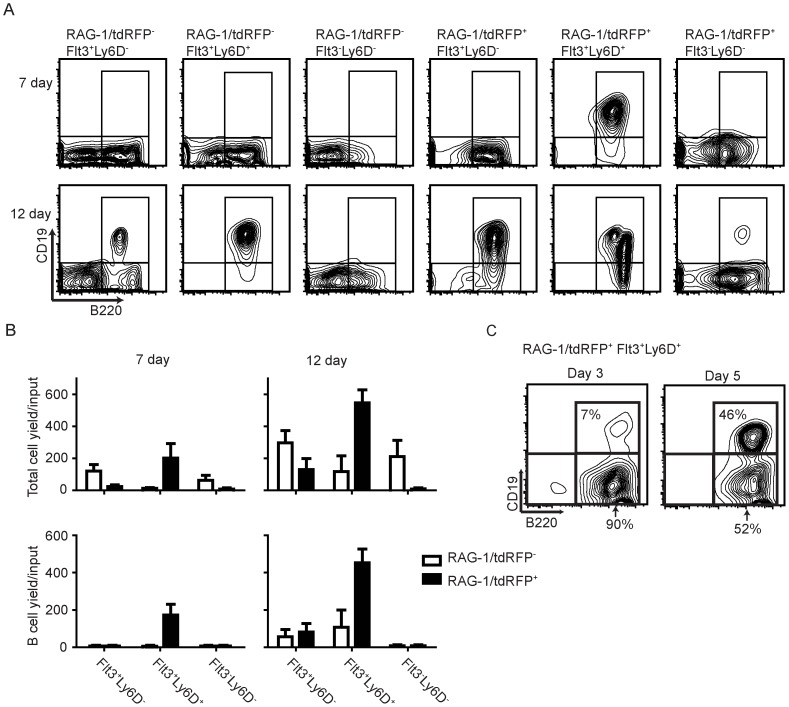
B lymphopoietic potential of CLP subsets. 1000 to 1500 cells of each of the CLP subsets gated as in Figure 1 were sorted and cultured under stromal cell free, serum free conditions in the presence of SCF, Flt3 ligand and IL-7. Cells were analyzed after 7 and 12 days of culture. (**A**) Flow cytometry profiles are shown for cells recovered from cultures. Viable B lineage cells were defined as B220^+^ CD19^+^. (**B**) Total cell and B lineage cell yields per input progenitor cell were calculated. The bars represent average yield ±SEM of five independent experiments. (**C**) Flt3^+^ tdRFP^+^ Ly6D^+^ CLPs as described in Figure 1A were sorted and cultured in stromal cell free, serum free medium with SCF, Flt3 ligand and IL-7. Cells were harvested and analyzed after 3 or 5 days of culture. The data is from one of three independent experiments that gave similar results.

The two Flt3^-^ subsets displayed some heterogeneity with respect to c-Kit, IL-7Rα, CD24, CD43, CD27 and CD93/AA4.1 expression (Figure 1B). However, these categories included few functional lymphoid progenitors (see Figure 2, below) and were not subdivided further.

The density of c-Kit can be highly informative about degree of maturation, and CLPs were originally defined as c-Kit^Lo^ [5], [6], [21], [26]. Therefore, the four Flt3^+^ subsets were arranged in Figure 1B according to median c-Kit fluorescent intensities. The density of IL-7Rα tended to be reciprocal to c-Kit, and higher on cells with a history of RAG-1 expression. All four subsets had high levels of CD24, a marker extensively used by Hardy and colleagues to characterize later stages of B lineage progression [1]. CD43 and CD93/AA4.1 may decline or increase, respectively with differentiation [1], as suggested by differences in their densities.

The Sca-1 member of the Ly6 family defines stem/progenitor cells in C57BL/6, but not BALB/c mice [27]. However, we found that Ly6D staining profiles for CLPs were indistinguishable in the two strains (data not shown).

We conclude that the CLP category can be reproducibly resolved into a minimum of six phenotypic subsets. Four of them could not be sub-divided further on the basis of five commonly used markers. Average percentages of total CLPs are given in the respective quadrants of Figure 1A and also shown in Figure S1.

### B lymphopoietic potential of CLP subsets

Of particular importance was the capability of CLPs to generate CD19^+^ lymphocytes in a given time. The two Flt3^-^ CD93^-/Lo^ categories of progenitors might be functionally insignificant, because each cell gave rise to less than 1 CD19^+^ CD45R/B220^+^ lymphocytes in defined 12-day cultures (Figure 2B and Figure S1). At the other extreme, Flt3^+^ tdRFP ^+^ Ly6D^+^ CLPs yielded over 400 lymphocytes per input cell. Figure S1 depicts a comparison of the relative abundance (shown in Figure 1) and yields per input progenitor in 12 day cultures (shown in Figure 2A). Similar conclusions were made about the lymphopoietic potential of the four subsets when they were assessed with limiting dilution assays (Figure S2).

The fractions likely differ with respect to degrees of lymphoid lineage progression, and the potent RAG-1/tdRFP^+^ Ly6D^+^ Flt3^+^ subset of CLPs was the only one to reproducibly generate significant numbers of B lymphocytes within one week (Figure 2A, 2B and Figure S1). Rapid acquisition of B lineage markers is another indication that this represents a relatively late stage of differentiation (Figure 2C). That is, all acquired CD45R/B220 within three days and half expressed CD19 by five days. The remaining three subsets produced lymphocytes in longer, 12 day cultures (Figure 2B). RT-PCR assays were then performed with selected genes that are required for B lymphopoiesis. As might be expected, the potent Flt3^+^ tdRFP^+^ Ly6D^+^ CLPs had high levels of RAG-1 transcripts (Figure S3). Affiliation with the B lineage was also reflected in their expression of Ebf-1 and Pax5. Ebf-1 and Pax5 are essential for B lymphopoiesis and high levels of Pax5 are needed to drive progenitor cells differentiation into B lineage cells expressing B220, CD19 and IgM [28]. Reciprocally, that subset and others with B potential, lacked transcripts for the Id2 transcription factor that is needed for NK lineage progression. Primitive CLPs marked by slow B lymphocyte generation and lack of lineage restriction had very low levels of RAG-1, Ebf-1 and Pax5 transcripts.

For reasons that are unclear, CLPs generate B lineage cells more efficiently in stromal cell-free cultures than in stromal cell co-cultures [29]. However, the latter conditions could be more supportive of earlier progenitors [30], so all six CLP subsets were plated on OP9 stromal adherent layers (Figure S4). Low numbers of CD19^+^ lymphocytes were only generated from the Flt3^+^ Ly6D^+^ RAG-1/tdRFP positive subset. The co-culture model did support more non-B lineage differentiation from other CLP subsets (see below).

A significant number of CLPs lacked Flt3 and Ly6D. While a minority of these cells had a history of RAG-1 expression, they lacked RAG-1 transcripts (Figure S3) and probably contribute little to B lymphopoiesis (Figure 2). Their low B lineage yields in 12 day cultures suggest they are either extremely primitive or developmentally arrested (Figure 2A and B). Ones expressing high levels of the IL-7Rα cytokine receptor would include primitive Flt3^-^ c-Kit^int/-^ Il-7Rα^Hi^ CD122^-^ NK lineage restricted progenitors [10], [11]. Thus, expression of RAG-1, Flt3 and Ly6D separately and collectively denote a major pathway(s) of B lymphopoiesis.

### Two CLP fractions have most of the non-B lineage differentiation potential

**Figure 3:**
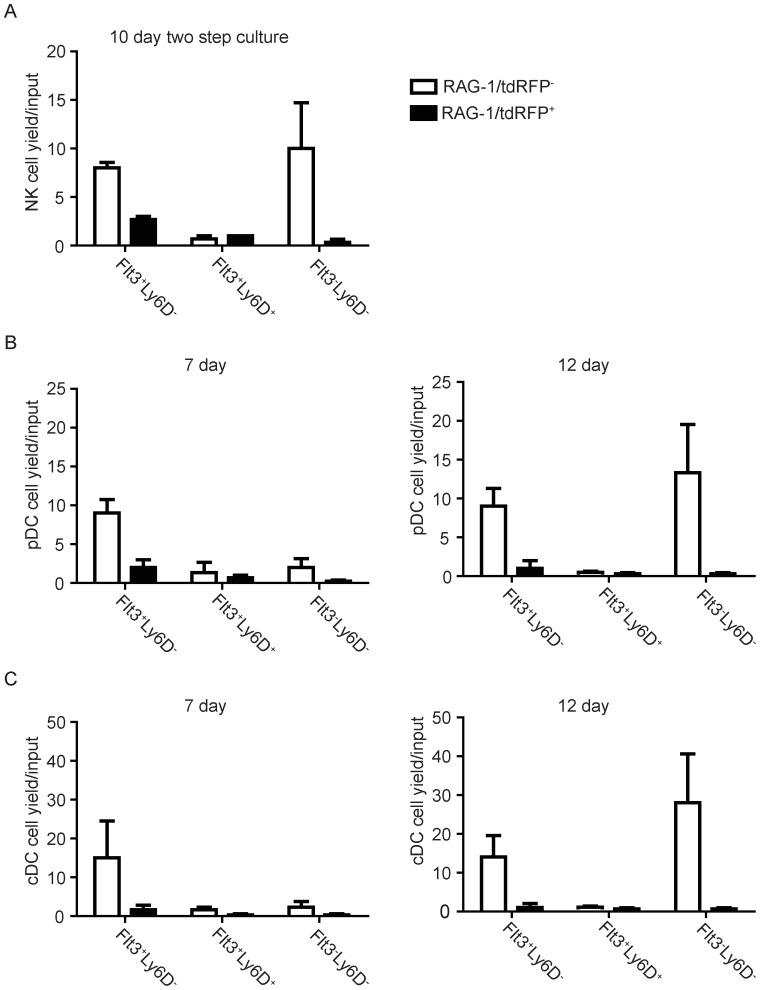
Two RAG-1/tdRFP^-^ CLP fractions have most of the non-B lineage differentiation potential. The six CLP subsets as gated in Figure 1 were sorted and cultured under stromal cell free, serum free conditions. Cells were cultured in the presence of SCF, Flt3 ligand and IL-7. Cells were harvested and analyzed after 7 or 12 days. (**A**) Two step stromal free, serum free cultures optimal for NK cell development were performed as indicated in Material and Methods. NK cells were gated as NK1.1^+^. (**B**) Plasmacytoid dendritic cells (pDCs) were defined as CD11c^Lo/+ = ^220^+^ Ly6C^+^ CD19^-^. (**C**) Conventional dendritic cells (cDCs) were defined as CD11^Lo/+^ CD11b^+^ B220^-^CD19^-^. Average yields ±SEM of at least three independent experiments are shown.

Lineage choice options become progressively restricted, and we assessed competency to produce other cell types (Figure 3). NK cell yields were evaluated using two-step cultures optimized for their generation. Other measurements were made with the same cultures illustrated in Figure 2, so conditions were optimized for B lymphopoiesis. Nonetheless, two of the six CLP subsets generated NK1.1^+^ NK cells, CD45R/B220^+^ CD11c^Lo/+^ Ly6C^+^ plasmacytoid dendritic cells (pDC) and CD45R/B220^-^ CD11c^+^ CD11b^+^ conventional dendritic cells (cDC) (Figure 3A, B and C). Small numbers of F4/80^+^ macrophages were also made (not shown). Neither of these CLP subsets had a history of RAG-1 expression, and both were Ly6D^-^. Potential to generate non-B lineage cells was more obvious when CLPs were co-cultured with OP-9 stromal cells (Figure S4). For example, both Flt3^-^ CLP subsets generated many NK cells. Interestingly, only RAG-1/tdRFP positive CLPs gave rise to NK cells marked by a history of RAG-1 expression (Figure S4).

Four subsets had T lineage lymphoid potential in OP9-DL1 co-cultures (Figure S5A), and that tended to correlate with relatively high levels of Notch (Figure S5B). However, the OP9-DL1 culture system is known to allow T lineage progression of many marrow fractions [31]. That is shown here to be the case for B lineage restricted CLP that express Ly6D [9]. CCR7 and CCR9 are chemokine receptors responsible for marrow progenitor migration to the thymus [32]-[35]. They are normally expressed through MPP and CLP stages [33], [36]. Real-time PCR revealed that CLPs with a history of RAG-1 expression had abundant chemokine receptor transcripts (Figure S5B).

The primitive nature of RAG-1/tdRFP^-^ Flt3^+^ Ly6D^-^ CLPs was also reflected in the long time required to make lymphocytes (Figure 2A and B), and we can now conclude that they are not lineage restricted. Ly6D acquisition denotes loss of in vivo T lineage differentiation potential [9]. Again, the Flt3^-^ Ly6D^-^ fractions had no T potential, high levels of Id2 and low levels of Notch. They likely overlap with Flt3^-^ CLPs described in a very recent study [37] and include the recently described pre-NKP [10], [11].

### Non-hierarchical acquisition of RAG-1 and Ly6D with B lineage progression

**Figure 4:**
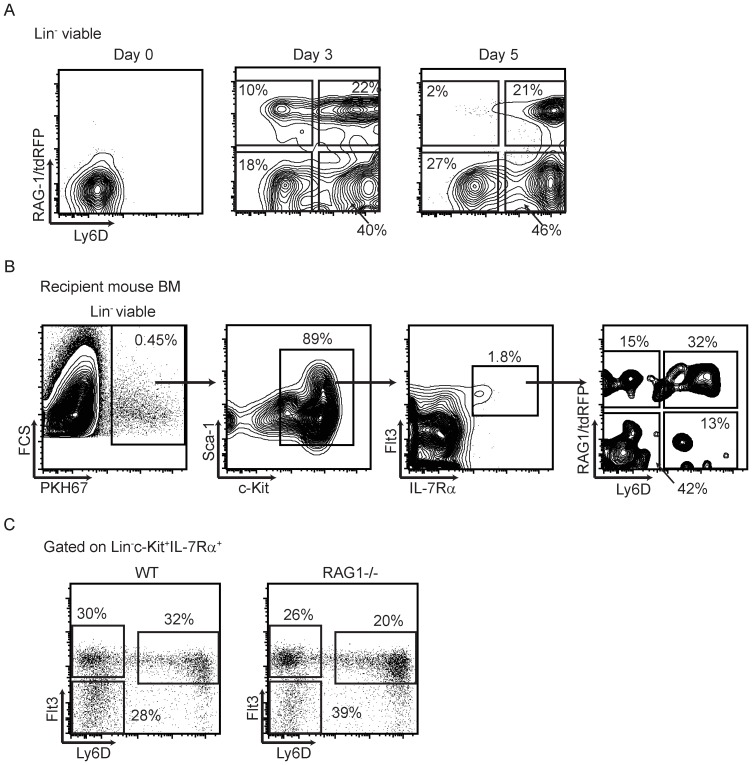
Non-hierarchical acquisition of RAG-1 and Ly6D with B lineage progression. (**A**) Flt3^+^ RAG-1/tdRFP^-^ Ly6D^-^ CLPs were sorted (top panels) and cultured under stromal cell free, serum free conditions in the presence of SCF, Flt3 ligand and IL-7. Cells were harvested and analyzed for the expression of Ly6D and RAG-1/tdRFP after 3 or 5 days of culture to assess lineage progression. (**B**) Primitive IL-7Rα^-^ RAG-1/tdRFP^-^ Ly6D^-^ LSK cells were sorted from RAG-1\Red mouse bone marrow, labeled with PKH67 fluorescent dye and transferred i.v.to non-irradiated WT recipient mice. Bone marrow was recovered 8 days later analyzed to determine progression through CLP subsets. (**C**) CLP subsets and percentages in WT and RAG-1 deficient mice. In each case, one representative of three independent experiments is shown.

The above findings suggest that Flt3^+^ RAG-1/tdRFP^-^ progenitors lacking Ly6D likely give rise to the highly potent Flt3^+^ RAG-1/tdRFP^+^ Ly6D^+^ CLPs. An issue was whether the RAG-1 locus is activated in those cells before or after expression of Ly6D. To address that question, we sorted Flt3^+^ RAG-1/tdRFP^-^ Ly6D^-^ CLPs and monitored their phenotypes with time in culture (Figure 4A). To our surprise, some of these CLPs became RAG-1/tdRFP^+^ before displaying Ly6D, while the reverse sequence was followed with other CLPs. However, in either situation, B cell precursors that are RAG-1/tdRFP^+^Ly6D^+^ were generated (data not shown). Real time PCR analysis revealed that they had elevated Ebf-1 transcripts consistent with lineage progression (data not shown). Furthermore, both differentiation patterns were observed when the stem/progenitor rich RAG-1/tdRFP^-^ LSK fraction of bone marrow was transferred to non-irradiated recipient mice (Figure 4B). We then examined bone marrow of RAG-1 knockout mice for possible linkage between RAG-1 and Ly6D. Percentages of total CLPs that expressed Ly6D were unaffected by loss of RAG-1 (Figure 4C). The same was true for patterns of Flt3 expression. Note that RAG-1/GFP reporter mice were used for this evaluation and no perturbation of any CLP subset resulted from homozygous RAG-1 deletion. RAG-1 history and Ly6D display are each powerful indicators of B lymphopoietic potential. However, the two are independently expressed and not coupled with respect to timing. Thus, there are at least two sequences for progression to the most lineage-restricted and productive of CLP states.

### CLP numbers are subject to sex steroid regulation

**Figure 5:**
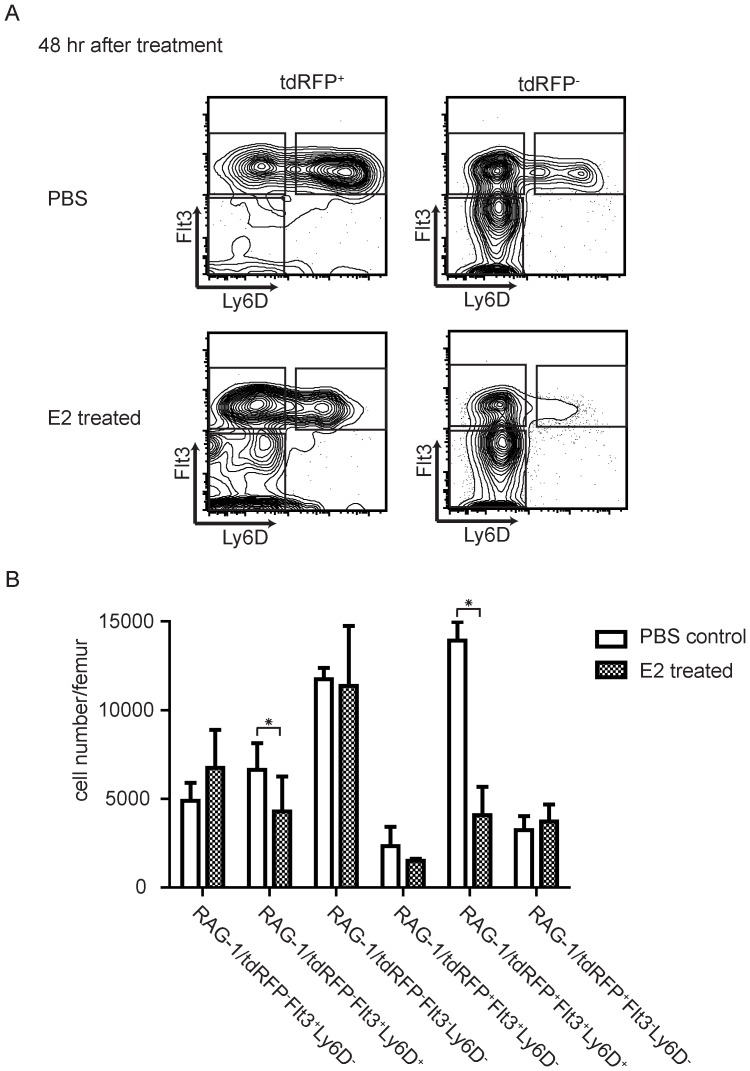
CLP subsets showed different sensitivity to estrogen induced deletion. Each RAG-1/Red mouse (n = 2 in each group, total three independent experiments) was injected with 1mg water soluble estrogen i.p. or PBS as control. Bone marrow was harvested and analyzed 12hr, 24hr, 48hr and 96hr after injection. Data shown is based on analysis of 48hr after injection. (**A**) FACS profile of control and estrogen-treated mice. CLP subsets were gated the same as shown in Figure 1. (**B**) Absolute numbers of each CLP subset from each mouse femur were calculated. Statistical analysis was done using the paired *t* test. Differences were considered statistically significant if *p*<0.05 (*).

ELP were originally defined in part by their sensitivity to estrogen, and B lymphopoiesis is likely to be regulated by steroid hormones [17], [38]. Lymphoid progenitors can be both direct and indirect hormone targets, complicating a full understanding of regulatory mechanisms [39], [40]. However, acute hormone treatment can be an effective experimental tool, suggesting relatedness of progenitor subsets. Therefore, we gave mice a single 1mg dose of water soluble estrogen and monitored changes in CLP subsets over time (Figure 5). The most significant changes occurred 48 hours later, when two Flt3^+^ Ly6D^+^ RAG-1/tdRFP^+/-^ subsets were significantly depleted (Figure 5). This was particularly the case for the abundant, highly B lymphopoietic Flt3^+^ Ly6D^+^ RAG-1/tdRFP^+^ CLPs, and they had largely recovered by 96 hours after treatment. The two populations likely derive from related, hormone sensitive progenitors, consistent with previous demonstrations that they are similarly lineage restricted [7], [9]. In contrast, we found hormone insensitivity of two Flt3^-^ CLP subsets, one or both of which include NK progenitors [10], [11].

## Discussion

There has been extensive debate about the potential of CLPs to generate B and T lymphocytes, dendritic and non-lymphoid cells, prolonged in part because many CLP definitions have been used [6], [8], [9], [19], [20]. It has been clear for some time that the originally described CLPs are heterogeneous [6]. For example, they are divisible according to RAG gene expression and recombinase activity as well as Flt3 and Ly6D display [9], [13], [14], [41]. However, it was uncertain if all populations belong in a single differentiation pathway and if there is a rigid order of marker acquisition. We exploited seven previously used parameters to reproducibly isolate six CLP subsets. While four of these are relatively homogeneous populations, our findings suggest they can acquire differentiated characteristics at least two ways. To be exact, expression of RAG-1 and Ly6D are both important, but unlinked milestones. One of the six subsets is lineage restricted and poised to generate large numbers of CD19^+^ lymphocytes. This information should be helpful in achieving consensus about the most favored of alternate differentiation pathways.

Although CLPs were originally defined by Kondo and colleagues as Lin^-^ c-Kit^lo^ IL-7Rα^+^, we studied a somewhat larger category of Lin^-^ c-Kit^lo/+^ IL-7Rα^+^ cells. This is because IL-7Rα is the one common feature of all CLP definitions, and improved sensitivity of detection has revealed a small overlap with LMPPs/ELPs. That is, small numbers of IL-7Rα^+^ are found among the Lin^-^ c-Kit^Hi^ fraction. This strategy did not include all categories of lymphoid progenitors. For example, B1 progenitors, lymphoid biased progenitors (LBP) and common lymphoid progenitor 2 (CLP-2) either express lineage associated markers or were not identified on the basis of IL-7Rα [24], [25], [42], [43]. However, the available information suggests these generate few lymphocytes or are downstream of the CLPs we studied here [24], [25], [42], [43].

Our findings confirm and extend many previous descriptions of B lineage progenitors [7]-[9], [14], [19], [20], [23]. Three out of seven parameters were particularly effective for sub-dividing the Lin^-^ c-Kit^lo/+^ IL-7Rα^+^ CLP fraction, but had to be used in combination to identify the most potent B lymphopoietic cells. Specifically, simultaneous display of Flt3 and Ly6D as well as a history of RAG-1 expression was characteristic of B lineage progenitors that could most quickly acquire CD19 in culture. The same Flt3^+^ Ly6D^+^ RAG-1^+^ subset, representing approximately 25% of all CLPs, was a poor source of NK and dendritic cells. Indeed, this was the case even when exposed to the TLR ligand LPS (unpublished observations). We consider them to be the most differentiated of all CLPs, and they must represent a major intermediate in B lymphopoiesis (Figure S6). Ly6D bearing CLPs were previously demonstrated to lack myeloid, NK and T lineage potential in vivo [9], [23], but we now show that expression of RAG-1 corresponds to greatly increased potency in generating B lineage cells. That fraction must include an even smaller subset of CLPs that were identified with a 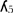 expression reporter [7], [15].

At the other extreme, approximately the same numbers of CLPs are Flt3^+^, but lack Ly6D and RAG-1. Their low transcripts for Ebf-1 as well as their slow differentiation and relative lineage instability suggest this may be the most primitive of CLPs [9], [15]. The transition between early and late CLPs appears not to be hierarchical, with some acquiring Ly6D before RAG-1 and others acquiring RAG-1 before Ly6D. Our culture and transplantation assays could not exclude a third possibility, that some Flt3^+^ CLPs simultaneously acquire both Ly6D and RAG-1. It is interesting that the Ly6D before RAG-1 sequence is more estrogen sensitive than alternative routes (see Figure 5). Thus, environmental changes may favor one over the other. Expression of Ly6D is IL-7 dependent, but occurs in Ebf-1^-/-^ as well as in RAG-1 knockout mice, again suggesting no rigid linkage between these events (Figure 4 and [23].

Approximately 30% of CLPs lack Flt3 as well as Ly6D and can be further subdivided on the basis of our RAG-1 reporter. The RAG-1^-^ subset has low CD93, high amounts of CD24 and is heterogeneous with respect to densities of c-Kit and IL-7Rα. While able to generate NK and dendritic cells, they do not acquire RAG-1 or generate B lineage cells in culture. Some of those CLP have a high density of IL-7Rα and therefore overlap with recently described pNKP [10], [11]. Because activated NK cells can bear CD11c [44], [45], it is noteworthy that we enumerated conventional dendritic cells on the basis of CD11c and CD11b co-expression. The RAG-1^+^ cohort partially resemble Lin^-^ Sca-1^+^ c-Kit^-^ lymphoid biased progenitors in having low c-Kit levels, high IL-7Rα, high CD93, high CD27 and high CD24 [24], [25]. Both of these CLP subsets express the Id2 transcriptional repressor, possibly explaining their limited ability to generate CD19^+^ lymphocytes.

Many fractions of bone marrow cells generate T lineage lymphocytes in OP9-DL1 stromal cell co-cultures [31], [46], [47], and that was particularly the case for the Flt3^+^ CLP subsets tested in this study. However, this model is not informative about which progenitors are likely to replenish the thymus [48], and we found that B lineage restricted CLPs bearing Ly6D [9] produced T lineage cells in this co-culture system. The low abundance of some CLP subsets represents a technical challenge for assessing their importance in more relevant *in vivo* assays [8], [49], [50]. As another long-term goal, it would be helpful to find a cell surface marker or marker combination that is as effective as RAG-1reporters for defining potent lymphoid progenitors.

It is now possible to resolve many subsets of stem/progenitors from hematopoietic tissues. While this reflects intrinsic heterogeneity and lack of synchrony of differentiation events, it seems unlikely that all differences in populations have biological significance. That requires consideration of their abundance in bone marrow, as well as the yields and time required to generate lymphocytes. Given that, we were surprised to find four CLP subsets with significant potential to generate lymphocytes. Patterns of gene and marker expression suggest relationships between these cells and two subsets appeared to converge on a relatively homogeneous progenitor of Pro B cells. It remains to be seen if the sequence of acquisition of differentiated characteristics correlates with important differences in the lymphocytes. Namely, do particular CLPs generate functionally specialized cells? A recent study revealed differences in CLPs generated from different HSC populations [51], suggesting intrinsic and inheritable divergence in HSC and progenitor subsets.

Most of these analyses were performed with normal young mice under steady state conditions, but B lymphopoiesis is known to be heavily influence by inflammation, hormonal changes and aging [21]. As one example, we found that the most abundant and potent of CLP subsets was transiently depleted following acute elevation of estrogen levels. Thus, that subset must be closely related to a hormone regulated stage.

NK lineage cells in bone marrow appear to derive from hormone regulated progenitors and are depleted by chronic delivery of estrogen in time-release pellets [52]. However, it is interesting that the Flt3^-^ pNKP containing fractions of CLPs were unaffected by a single bolus treatment of estrogen. That could mean that they have a relatively slow turnover rate, and no close affiliation with the most B lymphopoietic of CLPs.

Recombinase activity and D-J rearrangements are detectable in a substantial number of NK lineage cells in bone marrow [13]. However, pNKP expressed very little RAG-1/GFP reporter, consistent with our real-time PCR analyses [10] and Figure S3). This might also argue that NK lineage cells are slowly replaced under normal circumstances.

Studies with experimental animals provide useful background for learning about the transition of human HSC to lymphocytes [53]-[55]. The RAG-1/tdRFP^+^ Flt3^+^ Ly6D^+^ murine CLP subset may be equivalent to CD34^+^ CD10^Hi^ B lymphocyte progenitors in human bone marrow, which have less dendritic cell potential and express higher levels of Ebf1, Pax5 and RAG-1 than the CD34^+^ CD10^Lo^ progenitors [56]. Thus, it is unlikely that early steps in lineage restriction occur in abrupt, discontinuous steps, and via a single differentiation pathway. Eventually, relatively homogeneous cells accumulate that are poised to replenish the humoral immune system. While the process is surprisingly complex, we should become better able to pinpoint places where malignant transformation or immunodeficiencies occur.

## Materials and Methods

### Mice

RAG-1/Cre mice were backcrossed to the C57BL/6 background for at least nine generations. RAG-1/Red mice were created by crossing RAG-1/Cre (C57BL/6 background) with tdRFP knocked into the Rosa26 locus (C57BL/6, B6 background)[14]. Wild-type C57BL/6 mice were used as negative controls. Homozygous RAG-1/GFP mice on the C57BL/6 background were used as RAG-1 deficient mice. Balb/c mice were purchased from the Jackson Laboratory (Bar Harbor, ME).

### Ethics Statement

This study was carried out in strict accordance with the recommendations in the Guide for the Care and Use of Laboratory Animals of the National Institutes of Health. All animals were bred and maintained in the Laboratory Animal Resource Center at the Oklahoma Medical Research Foundation (Oklahoma City, OK). All experimental procedures were conducted under Institutional Animal Care and Use Committee Protocol K-0098-4, approved June 4, 2010, and annually thereafter, Animal Welfare Assurance # A3127-01.

### Isolation of cell populations and flow cytometry

Cell isolations were performed in PBS with 3% FCS. Marrow cells were harvested from the bones and erythrocytes were lysed by briefly re-suspending in NH4Cl^-^ hypotonic solution. Isolation of progenitor populations for culture was done as follows: bone marrow cells were enriched with negative selection by labeling marrow with Ly6G+C/Gr-1 (RB6-C5), CD11b/Mac-1, TER-119, CD3 (17A2), CD8 (53-6.7), CD19 (1D3), B220 (14.8), and then immunomagnetically depleted with the BioMag goat anti-rat IgG system (Qiagen, Valencia, CA). All cells were treated with FcR block (2.4G2) before staining. After staining with allophycocyanin-Cy7 (APC-Cy7) anti-lineage markers, APC anti-c-Kit (2B8), PE-Cy5 anti-Flk2/Flt3 (A2F10), Pacific Blue anti-Sca-1 (D7; eBioscience, San Diego, CA), biotin-streptavidin PE-Cy7 anti-IL-7Rα (A7R34; eBioscience) and FITC anti-Ly6D (49-H4), Lin^-^ populations were sorted using either a MoFlo (DakoCytomation, Carpinteria, CA) or FACSAria cytometer (BD Biosciences, San Jose, CA) into specific populations. Dead cells were excluded by propidium iodide (PI) staining (Molecular Probes, Grand Island, NY). Subset purification was confirmed by post-sort analysis. Isotype control were used for gating. For surface marker density analysis, lineage depleted bone marrow cells were stained with APC-Cy7 anti-lineage markers, Percp-Cy5.5 anti-c-Kit (2B8), PE-Cy5 anti-Flk2/Flt3 (A2F10), Pacific Blue anti-Sca-1 (D7; eBioscience), biotin-streptavidin PE-Cy7 anti-IL-7Rα (A7R34; eBioscience) and FITC or Alexa647 anti-Ly6D (49-H4), FITC anti-CD24 (M1/69) or FITC anti-CD43 (Ly-48) or APC anti-CD93 (AA4.1 eBioscience) or APC anti-CD27 (LG.3A10, Biolegend, San Diego, CA). All Abs came from BD Pharmingen (San Diego, CA), unless otherwise stated. Flow cytometry analyses were performed on a BD Biosciences LSRII. FlowJo software (Tree Star, Ashland, OR) was used for analysis.

### Estrogen treatment

Water soluble β-Estradiol (Sigma, St. Louis, MO) was dissolved in sterile PBS at 10mg/ml. 1mg β-estradiol (in 100 µl PBS) was delivered to each mouse through intraperitoneal injection. Each control mouse was given 100 µl PBS. Bone marrow cells were harvested after 48 hours and analyzed as indicated previously [17].

### Serum-free, stromal cell-free cell cultures

Detailed procedures are as described previously [40]. Briefly, sorted cells were cultured in 96-well plates (Corning, Union City, CA) with X-VIVO15 medium (Walkersville, BioWhittaker, MD) containing 1% detoxified BSA (StemCell Technologies, Vancouver, BC, Canada), 5×10^-5^M 2-ME, 2 mM L-glutamine, 100 U/ml penicillin, and 100 mg/ml streptomycin. Culture medium was enriched with 100 ng/ml Flt3 ligand (FL), 20 ng/ml stem cell factor (SCF), 1 ng/ml IL-7 or 50 ng/ml IL-15 in combinations as indicated. DC-lineage promoting conditions included SCF and FL enriched medium without IL-7. Two-step NK-lineage culture conditions have been described previously [52]. Briefly, cultures were initiated with CLPs in medium with SCF, FL, and IL-7. Cells were washed at day 5 and IL-7 was replaced by IL-15 for the remaining 5 days. Incubation was maintained at 37°C in a 5% CO2 humidified atmosphere. Cells were fed by replacing a half volume with fresh medium and cytokines every 3 or 4 days. Harvested cells were stained and analyzed on BD LSR II. Total cell yields were determined with Countbrite beads (Invitrogen) according to the manufacturer's protocol.

### In vitro cell labeling and adoptive transfer

CLP subsets were sorted and labeled with PKH67 Green Fluorescence using PKH67 Green Fluorescence Cell Linker Mini Kit (Sigma) using manufacturer's instruction. Labeled cells were injected intravenously into non-irradiated C57BL/6 mice. Bone marrow cells were harvested from recipient mice and analyzed eight days after transplantation.

### Statistics

The Prism 5.04 (Graphpad, La Jolla, CA) was used for statistical analysis. Values of *p* on a student *t* test were considered significant if <0.05.

## Supporting Information

Figure S1 The abundance and potency of B lymphocyte lineage progenitors in CLPs. The width of each pie slice is proportional to the average percentages that each CLP subset represents. The height of each pie slice is proportional to the average B lineage cell yield in 12 day cultures. The data used to prepare this graphic were separately shown above in Figures 1 and 2.
(TIF)

Figure S2 B lymphopoietic potential of CLP subsets. Indicated numbers (8 to 24 replicates per dilution) of cells from selected progenitor populations were sorted and cultured under stromal cell-free, serum-free conditions in the presence of IL-7, SCF and Flt3 ligand. Cells were harvested 13 days later and analyzed for B220^+^ CD19^+^ B lineage cell production.
(TIF)

Figure S3 The expression of lineage associated genes correlates with differentiation potential. The six CLP subsets were isolated as above, and then mRNA was extracted and used for real-time PCR. The transcript levels of indicated genes were normalized based on GAPDH expression, and shown as relative expression levels ± EM calculated from at least three experiments. The expression levels of the Flt3^+^ Ly6D^−^ RAG1/tdRFP^−^ subset was used as baseline (valued as 1) and the expression levels of other populations were calculated relative to that baseline.
(TIF)

Figure S4 Lineage potentials of CLP subsets in OP9 co-cultures. The six indicated CLP subsets (1500 cells per well) were sorted and cultured on monolayers of OP9 stromal cells in the presence of SCF, IL-7 and Flt3 ligand. Cells were harvested and analyzed after 7 or 12 days (data not shown) of culture. The data shown represents one of five independent experiments. (**A**) Cells were analyzed for B220^+^ CD19^+^ B lineage cell production. (**B**) Cells were analyzed for NK1.1^+^ NK lineage cell production.
(TIF)

Figure S5 T lineage potential of CLP subsets. (**A**) Sorted CLP subsets, MPP and thymic ETP were cultured on OP9-DL1 stromal cells in media containing Flt3 ligand and IL-7 for 7 and 12 days. Cells were harvested and analyzed for T lineage cell (CD45.2^+^ CD44^+^ CD25^+^) yield. (**B**) mRNA was extracted from CLP subsets, HSCs and thymic ETP and then used as templates for cDNA synthesis. Real-time PCR for the indicated genes was performed. The transcript levels of indicated genes were normalized based on GAPDH expression, and shown as relative expression levels ±SEM calculated from three experiments. The gene expression levels of HSC or Flt3^−^ Ly6D^−^ RAG-1/tdRFP^−^ CLPs were used as baselines.
(TIF)

Figure S6 Developmental model of CLPs. Using Flt3, Ly6D and RAG-1/tdRFP, we resolved CLPs into six subsets. The two Flt3^−^ subsets were heterogeneous and showed poor B lymphocyte lineage potential. They likely include recently described pNKP and, the Flt3^−^ tdRFP^−^ subset could differentiate into NK and DCs. Among the four Flt3^+^ subsets, cells lacking RAG-1 and Ly6D generated B lineage cells, NK and DCs, and they were also ancestors for the other three Flt3^+^ CLP subsets. Ly6D and RAG-1 each marked B lineage progression. However, they were expressed independently and asynchronously. The convergence of these two markers eventually labeled the most potent and immediate of B cell precursors. Major differentiation pathways are indicated by bold arrows, while dotted lines indicate areas that merit additional study.
(TIF)

Materials and Methods S1 (DOCX)
