# Supplementary figures and images for: RAG-1 and Ly6D Independently Reflect Progression in the B Lymphoid Lineage
Source: PLoS One. 2013 Aug 30;8(8):e72397. doi: 10.1371/journal.pone.0072397 (PMC3758291; doi:10.1371/journal.pone.0072397)

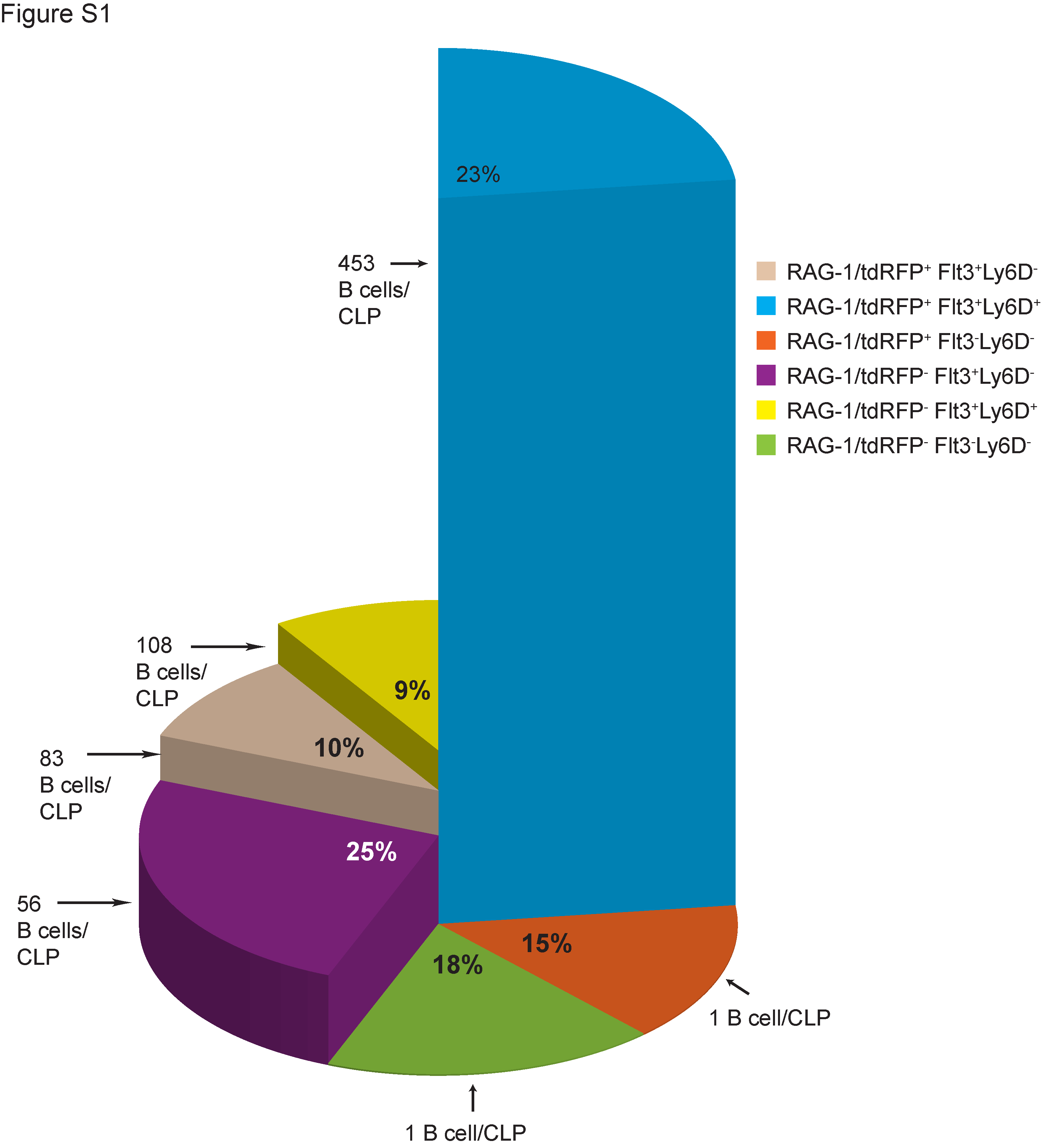

Supplement: Figure S1 — The abundance and potency of B lymphocyte lineage progenitors in CLPs. The width of each pie slice is proportional to the average percentages that each CLP subset represents. The height of each pie slice is proportional to the average B lineage cell yield in 12 day cultures. The data used to prepare this graphic were separately shown above in Figures 1 and 2. (TIF) [file pone.0072397.s001.tif]

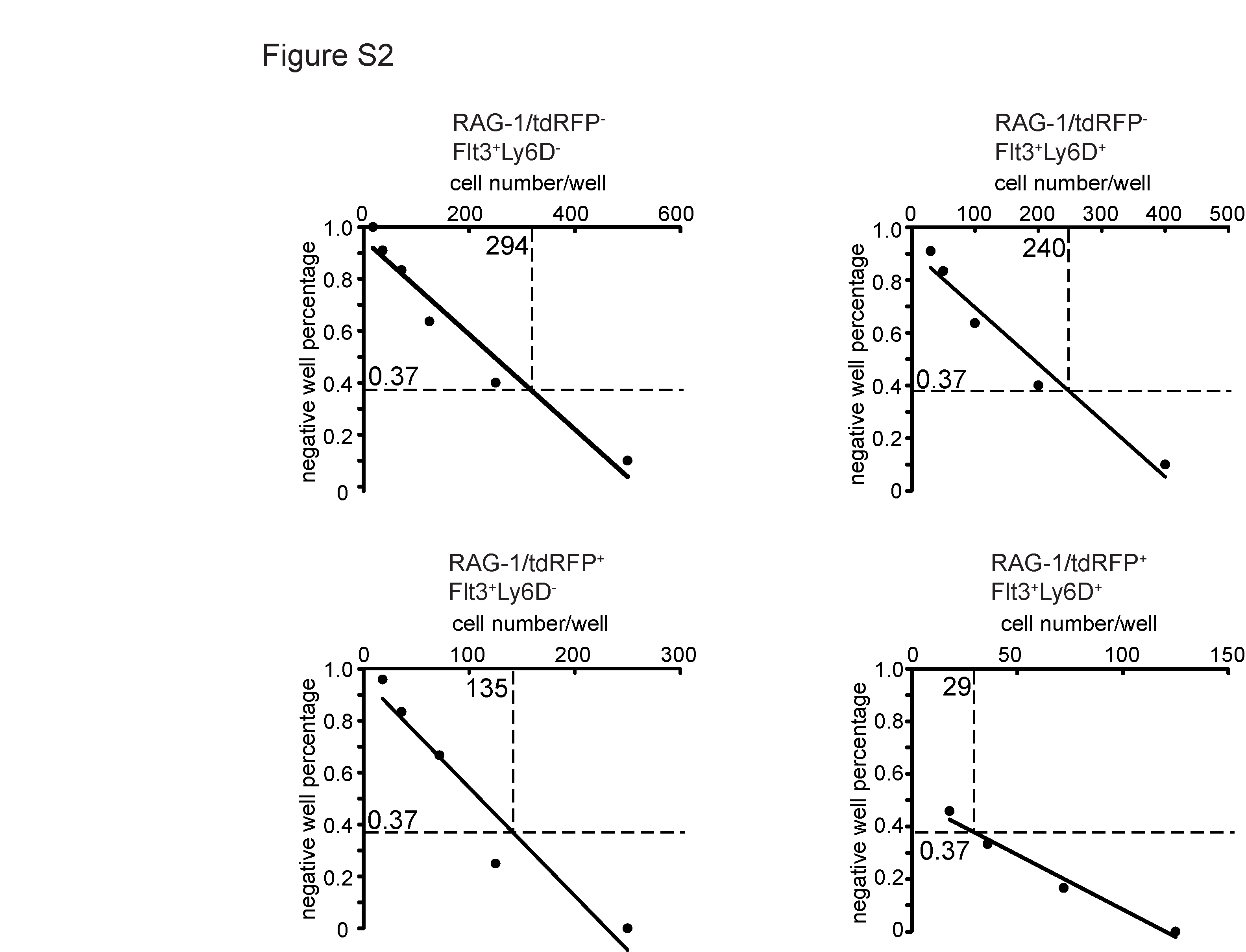

Supplement: Figure S2 — B lymphopoietic potential of CLP subsets. Indicated numbers (8 to 24 replicates per dilution) of cells from selected progenitor populations were sorted and cultured under stromal cell-free, serum-free conditions in the presence of IL-7, SCF and Flt3 ligand. Cells were harvested 13 days later and analyzed for B220+ CD19+ B lineage cell production. (TIF) [file pone.0072397.s002.tif]

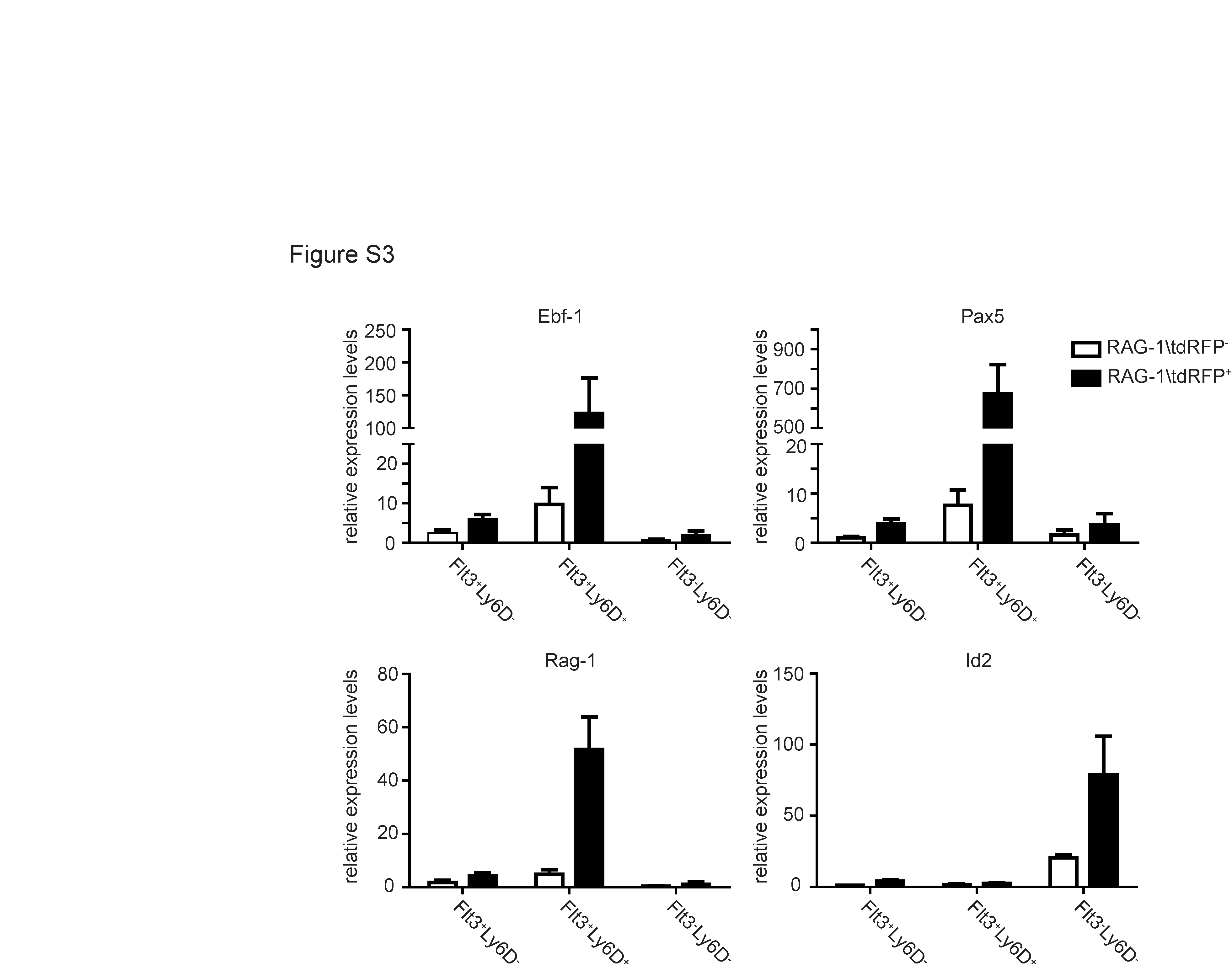

Supplement: Figure S3 — The expression of lineage associated genes correlates with differentiation potential. The six CLP subsets were isolated as above, and then mRNA was extracted and used for real-time PCR. The transcript levels of indicated genes were normalized based on GAPDH expression, and shown as relative expression levels ± EM calculated from at least three experiments. The expression levels of the Flt3+ Ly6D− RAG1/tdRFP− subset was used as baseline (valued as 1) and the expression levels of other populations were calculated relative to that baseline. (TIF) [file pone.0072397.s003.tif]

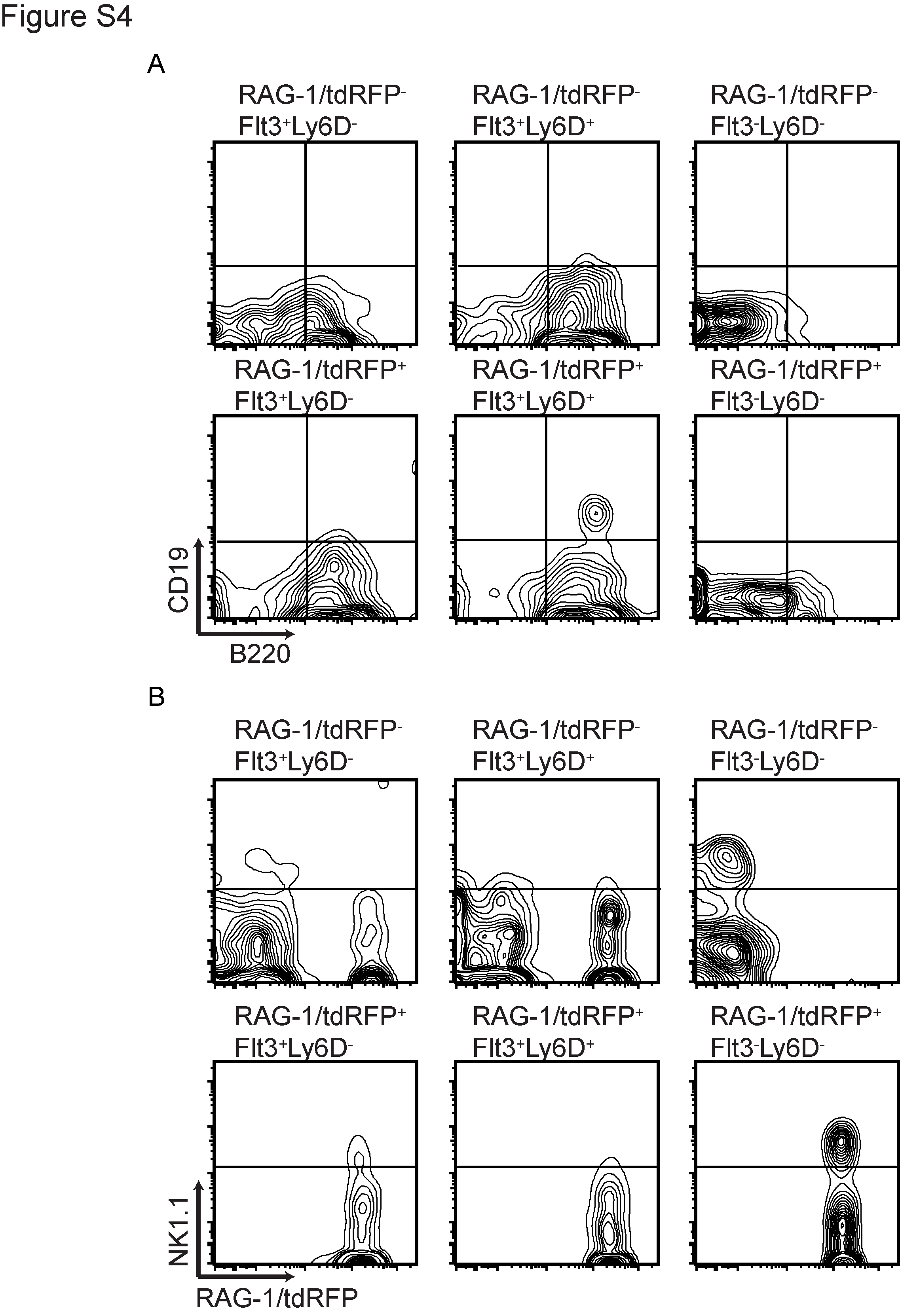

Supplement: Figure S4 — Lineage potentials of CLP subsets in OP9 co-cultures. The six indicated CLP subsets (1500 cells per well) were sorted and cultured on monolayers of OP9 stromal cells in the presence of SCF, IL-7 and Flt3 ligand. Cells were harvested and analyzed after 7 or 12 days (data not shown) of culture. The data shown represents one of five independent experiments. (A) Cells were analyzed for B220+ CD19+ B lineage cell production. (B) Cells were analyzed for NK1.1+ NK lineage cell production. (TIF) [file pone.0072397.s004.tif]

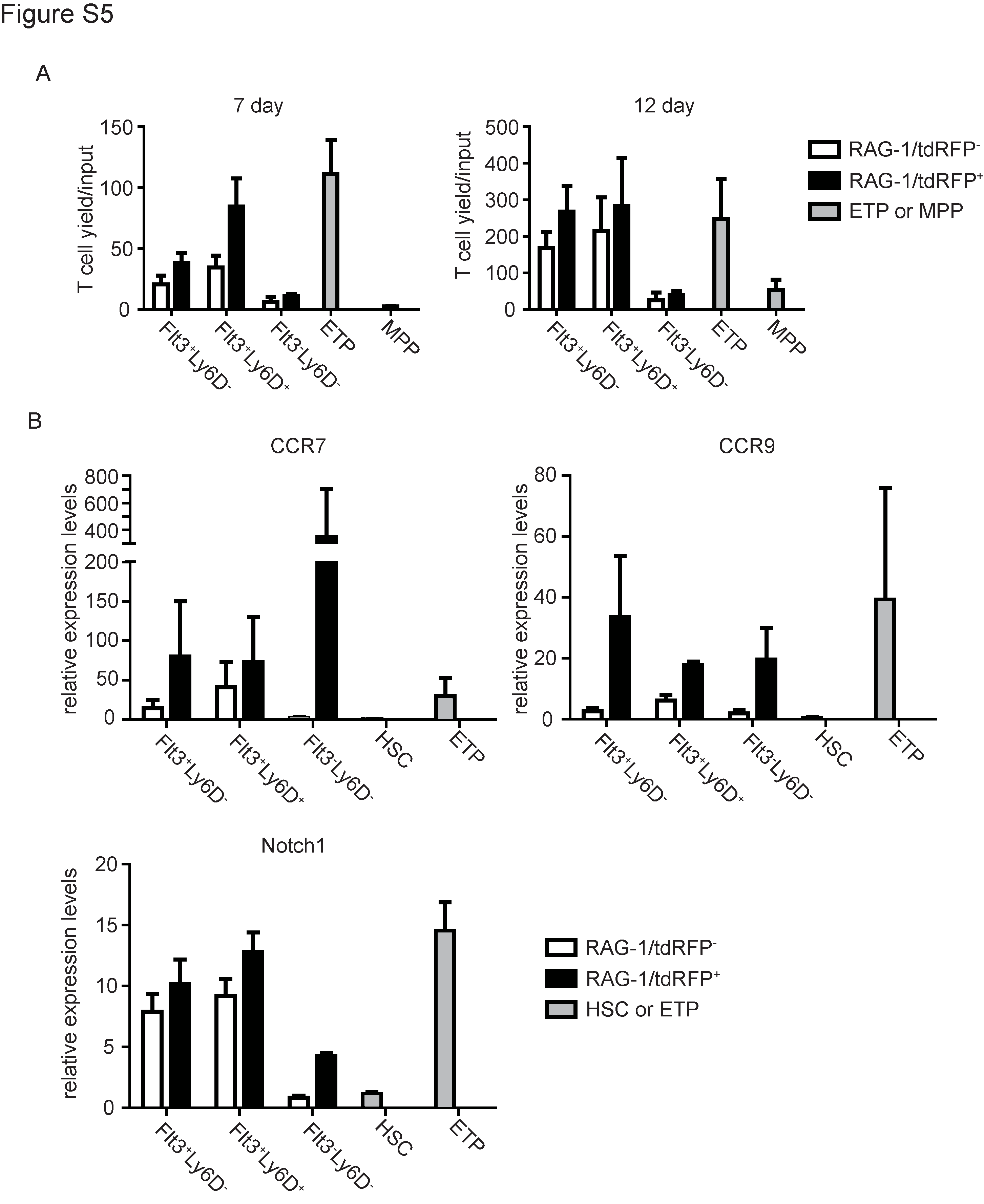

Supplement: Figure S5 — T lineage potential of CLP subsets. (A) Sorted CLP subsets, MPP and thymic ETP were cultured on OP9-DL1 stromal cells in media containing Flt3 ligand and IL-7 for 7 and 12 days. Cells were harvested and analyzed for T lineage cell (CD45.2+ CD44+ CD25+) yield. (B) mRNA was extracted from CLP subsets, HSCs and thymic ETP and then used as templates for cDNA synthesis. Real-time PCR for the indicated genes was performed. The transcript levels of indicated genes were normalized based on GAPDH expression, and shown as relative expression levels ±SEM calculated from three experiments. The gene expression levels of HSC or Flt3− Ly6D− RAG-1/tdRFP− CLPs were used as baselines. (TIF) [file pone.0072397.s005.tif]

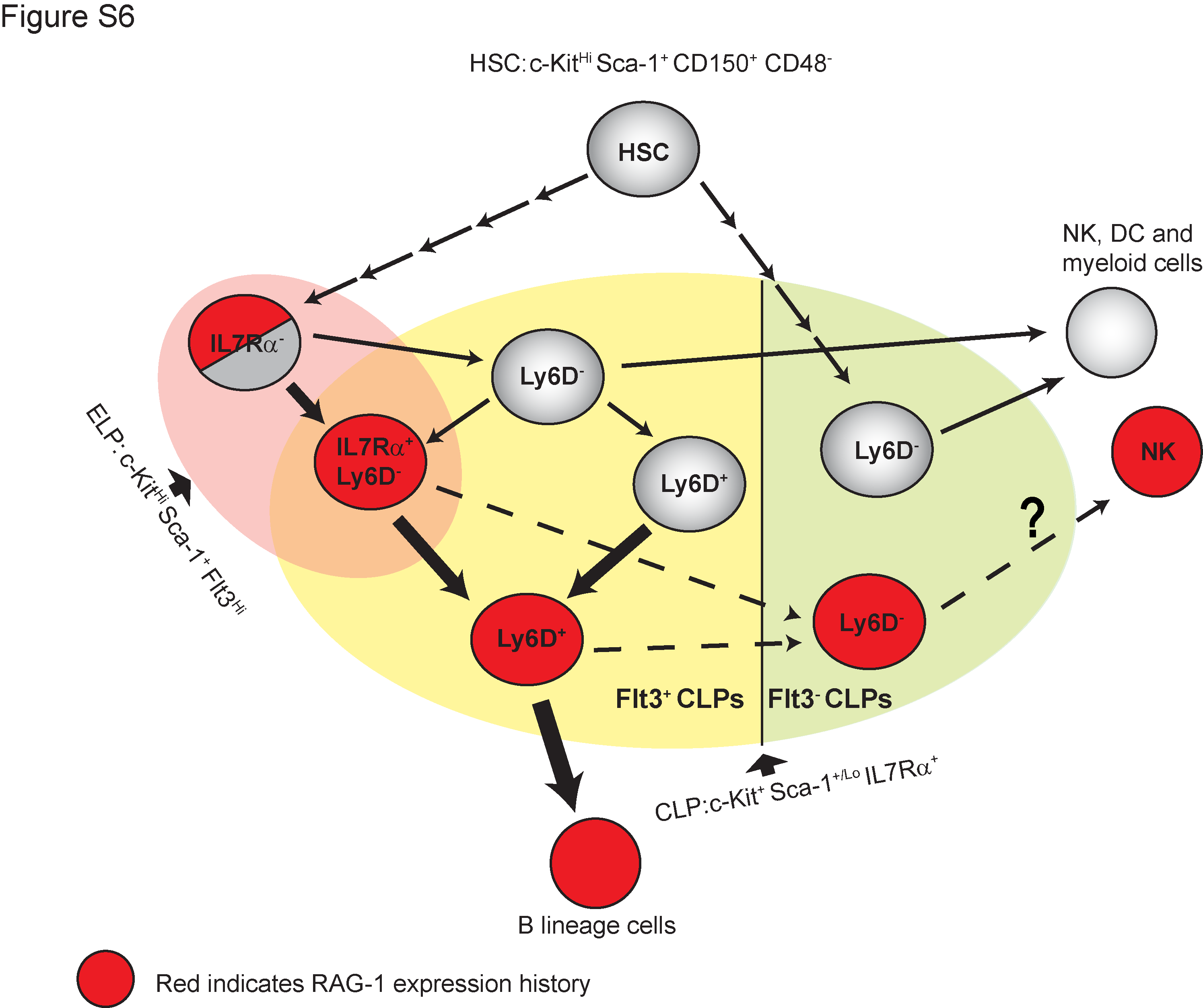

Supplement: Figure S6 — Developmental model of CLPs. Using Flt3, Ly6D and RAG-1/tdRFP, we resolved CLPs into six subsets. The two Flt3− subsets were heterogeneous and showed poor B lymphocyte lineage potential. They likely include recently described pNKP and, the Flt3− tdRFP− subset could differentiate into NK and DCs. Among the four Flt3+ subsets, cells lacking RAG-1 and Ly6D generated B lineage cells, NK and DCs, and they were also ancestors for the other three Flt3+ CLP subsets. Ly6D and RAG-1 each marked B lineage progression. However, they were expressed independently and asynchronously. The convergence of these two markers eventually labeled the most potent and immediate of B cell precursors. Major differentiation pathways are indicated by bold arrows, while dotted lines indicate areas that merit additional study. (TIF) [file pone.0072397.s006.tif]
