# Supplementary material for: RAG-1 and Ly6D Independently Reflect Progression in the B Lymphoid Lineage
Source: PLoS One. 2013 Aug 30;8(8):e72397. doi: 10.1371/journal.pone.0072397 (PMC3758291; doi:10.1371/journal.pone.0072397)
Supplement: Materials and Methods S1 — (DOCX) [file pone.0072397.s007.docx]

**Supplemental Material and Methods**

*Real-time quantitative PCR analysis of gene expression*

The mRNAs were isolated from sorted cells using TRIzol Reagent (Ambion, Life technologies, Grand Island, NY) and treated with DNase I. The cDNAs were then synthesized using Moloney murine leukemia virus reverse transcriptase (Invitrogen, Grand Island, NY). Reactions were quantified with the fluorescent TaqMan technology. TaqMan primers and probes specific for indicated genes were used in the ABI7500 sequence detection system using TaqMan Universal PCR Master Mix (Applied Biosystems, Carlsbad, CA). Reactions were run at an annealing temperature of 60°C with 40 cycles. Each sample was measured in triplicate, and the comparative threshold cycle method was used for relative quantification of gene expression. Relative fold changes were normalized to GAPDH expression level.

*OP9 and OP9-DL1 co-cultures*

The indicated populations were sorted and seeded onto monolayers of OP9 or OP9-DL1 stromal cells. Stromal cells were maintained in αMEM containing 10% FCS, 2mM L-glutamine, 5 x 10^-5^ M 2-mercaptoethanol, 100U/mL of penicillin and streptomycin. 20ng/mL FCS, 10ng/mL Flt3 ligand and 1ng/mL IL-7 were present in media for OP9 co-culture. 5ng/mL Flt3 ligand and 1ng/mL IL-7 were added to OP9-DL1 co-culture media. Cells were cultured for the indicated times.

*Limiting dilution assay*

Indicated numbers of cells were sorted into 96-well round bottom plates and cultured under stromal cell-free, serum-free conditions as described above. Cells were harvested after 13 days of culture, stained with monoclonal antibodies described above and analyzed on a BD FACS LSRII. The data was analyzed using Lcalc software (Stem Cell Technologies, Vancouver, BC, Canada).
